# Achieving external validity in home advantage research: generalizing crowd noise effects

**DOI:** 10.3389/fpsyg.2014.00532

**Published:** 2014-06-02

**Authors:** Tony D. Myers

**Affiliations:** Physical Education and Sports Studies, Newman UniversityBirmingham, UK

**Keywords:** home advantage, Muay Thai, internal validity, external validity, representative design

## Abstract

Different factors have been postulated to explain the home advantage phenomenon in sport. One plausible explanation investigated has been the influence of a partisan home crowd on sports officials' decisions. Different types of studies have tested the crowd influence hypothesis including purposefully designed experiments. However, while experimental studies investigating crowd influences have high levels of internal validity, they suffer from a lack of external validity; decision-making in a laboratory setting bearing little resemblance to decision-making in live sports settings. This focused review initially considers threats to external validity in applied and theoretical experimental research. Discussing how such threats can be addressed using representative design by focusing on a recently published study that arguably provides the first experimental evidence of the impact of live crowd noise on officials in sport. The findings of this controlled experiment conducted in a real tournament setting offer a level of confirmation of the findings of laboratory studies in the area. Finally directions for future research and the future conduct of crowd noise studies are discussed.

## introduction

The advantage gained by an athlete or team playing at their home venue is well-established in both popular culture and academic literature (Pollard and Pollard, 2005). Given that, in general, **home advantage** has proved ubiquitous and robust across team sports, subjectively judged individual sports and across time periods, the phenomenon has generated considerable theoretical and empirical interest from researchers. Different influences have been postulated and investigated including familiarity with the venue (e.g., Schwartz and Barsky, 1977; Pollard, 2002), travel and rule factors (e.g., Schwartz and Barsky, 1977; Courneya and Carron, 1992), hormonal responses in players (e.g., Neave and Wolfson, 2003; Carré et al., 2006), and crowd influences on both players and sport officials (Schwartz and Barsky, 1977).

KEY CONCEPT 1. Home advantage Advantage afforded to competitors and teams who compete at a home venue in front of supportive fans.

Sports fans themselves often consider their own influence paramount, feeling responsible for inspiring victory, distracting the opposition, and influencing officials (Wolfson et al., 2005). Interestingly, research suggests this view may not be entirely fanciful, with several researchers suggesting that the crowd could make an imposrtant contribution to home advantage. While there appears to be limited support for partisan fans actually positively influencing home players, there is some support for the jeers of a home crowd damaging away team performance (Epting et al., 2011). In addition to crowd influences on players' performance, researchers have also investigated the crowd effects on officials, with this area particularly fruitful in attempting to explain home advantage effects.

## Methods used to explore crowd effects on sport officials

Different methods have been used to explore crowd influences on officials: retrospective analysis of archival data; natural experiments; and specifically designed laboratory experiments. Archival data offers the possibility of analyzing large amounts of data spanning many years (e.g., Balmer et al., 2001). For example, Balmer and colleagues examined the results of all Winter Olympics events between 1908 and 1998 from a website that detailed medal totals and points for each country, in each Olympic event (e.g., Alpine skiing downhill men) across the different Olympic games. They were able to use this data to consider differences in home advantage effects of subjectively judged sports (e.g., judged by a panel) compared with those determined by an objective measure (e.g., time), as well as the influences of familiarity and travel factors. While exclusively relying on this type of evidence can be questioned given it is not collected specifically for purpose (with limits to the **internal validity** of findings), the evidence generated is useful when considered alongside specifically designed studies.

KEY CONCEPT 2. Internal validity The extent to which the causes of an effect are established by an inquiry.

In contrast, serendipity occasionally delivers a situation where is possible to observe the impact of “naturally occurring” crowd conditions, such as when teams have had to play without spectators present (e.g., Moore and Brylinsky, 1993; Pettersson-Lidbom and Priks, 2007; Van de Ven, 2011). For example, when Italian soccer was played for a period without spectators for safety reasons, Pettersson-Lidbom and Priks (2007) and more recently Van de Ven (2011), took the opportunity to compare home advantage and referee behavior in stadia with and without the usual crowds present. Pettersson-Lidbom and Priks found referees' adjusted their decisions to appease home supporters, punishing away players more harshly and treating home players more leniently. Interestingly, Van de Van found no difference in home advantage across the same series of games. This suggests that a crowd may influence officials' behavior but may be unnecessary for home advantage to occur. Such chance occurrences are attractive given their “real world” setting but are pre-experimental in terms of their research design with no deliberate manipulation of variables (Creswell, 2009). To demonstrate cause-and-effect specifically designed experiments are necessary.

Experiments with high internal validity have demonstrated the significant impact crowd noise has on influencing sport officials' decisions. For example, Nevill et al. (1999, 2002) and Balmer et al. (2007) conducted experiments where soccer referees made decisions in the presence of crowd noise (noise condition), or without crowd noise (a no noise condition). Both studies involved participants watching video footage 47 incidents each lasting approximately 9 s of an English Premier League soccer match (Liverpool vs. Leicester City) from the 1998/99 UK soccer season. In all of these experiments, the presence of crowd noise resulted in an imbalance of decisions in favor of the home side when compared to the “no noise” condition. In these studies the experimental designs used had high internal validity but we question aspects of their **external validity**, and particularly the extent to which this type of lab based experiment can be generalized to the real world.

KEY CONCEPT 3. External validity The extent to which one may safely generalize an inference (a) from the sample studied to the defined target population and (b) to other populations.

## External validity in crowd noise research

Those involved in research will be familiar with the concept of external validity. However, for those less familiar with different types of validity, the concept of external validity refers to the question of generalizability (Campbell and Stanley, 1966), specifically it refers to the degree to which observed causal relationships can be generalized across different participant groups, time periods, measures, and settings (Calder et al., 1982). The argument for high external validity is associated with not only knowing that the independent variable has a systematic impact on the dependent variable, but that this also likely to be the case in other settings and with different populations. Lynch (1982) explained this by discussing three aspects of external validity: statistical generalizability referring to the use of appropriate sampling methods to ensure results can be generalized to the larger population of interest; robustness—meaning the degree to which a cause-effect relationship found in an experiment can be replicated with different participants, contexts, and time intervals; and realism—referring to whether the research tasks, stimuli, and settings are realistic, the results being more likely to generalize to a natural environment.

Lynch (1999) argued the later aspect of realism was less important than other facets of external validity, arguing that the findings from studies using “real” people in “real-world” settings were no more likely to generalize than those from laboratory settings with student subjects. Nevertheless, a contrasting perspective on settings and their relationships to individuals was proposed by Brunswik (1952) in his concept of **representative design**. Simply put, representative design applies sampling theory to the input and environmental conditions of an experiment. Brunswik (1956) suggested that given individuals are never in reality independent of an environment, studies need to consider the environment and the person's interaction with it. As such, highlighting a need for environmental relations and any stimuli investigated being sampled from a natural environment to which the experimenter wishes to generalize (Brunswik, 1956). While representative design has generally failed to be integrated into the behavioral sciences, the relevance of applying this concept to sports psychology experiments has been argued for recently (Pinder et al., 2011). Using such an approach may help address the limitations of the laboratory settings highlighted by researchers in crowd noise research (Unkelbach and Memmert, 2010).

KEY CONCEPT 4. Representative design This concept refers to experimental designs that capture the salient features of any intended reality being investigated, including participants, tasks, and settings.

Nevill et al. (2002) claimed strong external validity for the findings of their study. This was based on the degree of similarity in decisions made by participants assigned to the noise group and those of the original match referees. This seems plausible, but in order to satisfy their claims of strong external validity, the research team needed to address a number of threats to external validity associated with measures, participants, and settings. The measurements used in the study do arguably address threats to external validity as they involved the type of decisions referees have to make in an actual game. Similarly, while the sample of 40 qualified referees from a referees club in England were volunteers, they do seem representative of a pool of referees as ranging in experience from newly qualified referees to 43 years of refereeing experience. This gives a level of confidence that the findings were generalizable across experience levels. The issue with external validity centers on two aspects of the study's setting. A major criticism of this study focused on the fact that the video footage used was taken from a single English Premier League soccer match (Liverpool vs. Leicester City). It is argued that the use of a single setting means it is not possible to determine if this is a more generalizable effect or one specific to this particular game (Sutter and Kocher, 2004; Unkelbach and Memmert, 2010). The other issue with the setting was its lack of representative design. Referees were making decisions in a comfortable laboratory setting with no consequence to their decisions. Clearly this does not replicate the reality of decision making in front of supporters and players that have outcome consequences.

Similar issues with external validity are evident in a study on crowd noise effects on judging decisions of **Muay Thai** officials (Myers et al., 2012). This study also used video with recorded crowd noise and no crowd noise as the independent variable manipulation. Participants were shown a Muay Thai fight videoed from the perspective of a judge from a single angle, projected onto a screen using a video-projection system. The eventual winner of the bout had the greatest vocal support with the greatest number of cheers over the course of the bout. Nevertheless, to attempt a representative design and replicate what is generally the case in an actual competition environment, the research team used footage where the losing competitor also had some crowd, albeit far less support. Again, the authors found a crowd noise and home advantage effect.

KEY CONCEPT 5. Muay Thai It is the national sport of Thailand that has recently grown in international popularity. It is a form of boxing that is competed in a standard boxing ring over five rounds, where gloved competitors kick, punch, knee, elbow, and grapple with their opponent using full-contact strikes in an attempt to stop their opponent or gain a points victory.

As with Nevill et al.'s (2002) study, the real threats to external validity in the Muay Thai study primarily include issues with the setting. The research team recruited 10 qualified and highly experienced Muay Thai judges from both the UK and Thailand. The number of participants was determined by a priori power analysis but the sample was again voluntary. While participants were from more than a single country, their experience varied less than those involved in Nevill et al.'s (2002) study. The issues with external validity centered on participant judges only being exposed to a single Muay Thai bout, making generalizability to other fights questionable. Moreover, while the realism of the task was acceptable, the stimuli and setting were not. Sitting in a comfortable laboratory, watching a video on a screen with recorded crowd noise with no consequence to any of the decisions made does not replicate the actual judging environment. This is an issue when the aim is to generalization to environments where such pressures exist.

Unkelbach and Memmert (2010) addressed one of the threats to the external validity associated with the setting by using video footage from 56 different soccer games together with recorded crowd reactions to fouls at different stadiums, showing participants footage of challenges with high and low volumes of crowd noise. The use of different scenes of challenges from different games and teams mean they were able to eliminate any possible team affect. The research team found a crowd noise effect although their results differed from those of Nevill et al.'s (2002) study, in that they found an increase the number of yellow cards awarded to the away team rather than fewer challenges awarded for the home team when crowd noise was present. Unkelbach and Memmert (2010) highlighted the realism of the setting as a limitation of their study, pointing out that referees who judge scenes on a video screen are hardly in the same situation as referees on the pitch, again acknowledging the likely impact of different environments on individuals' behavior. Essentially in these studies, we can be confident that the differences observed were a result of our intervention, but far less confident that similar differences would be observed in the real world.

## Enhancing external validity and representative design

In our recent study on the live crowd noise effects on Muay Thai judges (Myers and Balmer, 2012), we set out to address the threats to external validity evident in experimental crowd noise studies published to date by addressing issues of setting and representative design using multiple live settings. Using actual judges in real competition offers a number of advantages over and above the methodology employed in laboratory studies and relates to Brunswik's (1956) concept of natural stimulus and participant environment interactions. Using a live crowd and a live setting where judgments matter enhances the possibility of generalizing the results to similar live settings. Similarly, we felt using actual judges' scores at ringside that decide the actual outcome of competition means we can also begin to assess the practical significance of findings in a realistic way. This is something we felt has not been evident in previous experimental studies in this area. For example, the fouls identified by participants in previous studies have an undetermined impact on the outcome of a match and provide less credible evidence of practical significance. Our use of numerous bouts at multiple venues avoids the possibility of there being any localized effect, either associated with a particular venue or competitor. As such, avoiding a comparable “Liverpool effect” claimed by some to explain the findings of Nevill and colleagues (e.g., Sutter and Kocher, 2004).

Giving consideration to the possibility of generalizing to all Muay Thai judges, we used 17 qualified Muay Thai judges from England with varying experience. These judges ranged from newly qualified judges (*n* = 5), those with less than 3 years experience (*n* = 4) to those considered among the best in the UK with extensive experience of judging not only at national but also at major international shows (*n* = 8). With setting generalization in mind, the level of competition in our study varied in standard from international bouts involving elite competitors to more novice level bouts across 30 bouts.

Similarly we were mindful to capture different crowd factors, as such, crowd sizes varied from 500 to 3000, with proximity between judges and crowd varying from two to several meters. The composition of crowds also varied. For example, the majority of the crowd at the small hall shows used in the study comprised largely of friends of the fighters, those training in the sport, and fans that clearly understood rules and strategies. However, on the larger shows these spectator groups were joined by a large number of more general spectators less familiar with the sport.

While there are no dedicated stadiums for Muay Thai in the UK, the same venues do tend to be used regularly and so have what can be considered home fighters. In the study data was only used for bouts where there was a clear home fighter. Domestic home fighters were classed as such if they lived in the city or town where the venue was situated, had competed previously at the venue, and were matched against an “out of town” opponent. In international matches, the UK fighter was considered the home fighter and their foreign opponent the away fighter.

Four judges seated at ringside judged each of the thirty, five round bouts for which data were collected, with two judges randomized to the “crowd noise” condition and two to the “no crowd noise” condition. The “crowd noise” condition involved judges experiencing the natural crowd noise while situated at ringside, with judges in the no noise condition wearing noise cancelling headphones and listening to a track of white noise (leaving no perceptible crowd noise), also seated at ringside. The ringside judges scored each round of each bout using the actual scoring system applied in judging competitions, known as a “10-point must” system and identical to that used in professional boxing. While it is called a “10-point must” system, in practice it can be considered largely a binary scoring system with 10 points awarded to the winner and 9 points to the loser of a round. This is the case unless one competitor totally dominates or the referee is forced to count one of the competitors. In this case, point differences can be a large as 3 points difference in a single round. At the end of a bout each judge sums their scores for the five rounds to determine the winner (the higher score indicating the winner). For each bout, we recorded the judge's name, the condition in which they judged each bout, and the points they awarded each of the two boxers, together with a record of which boxer was the home competitor. This produced a total of 120 judgments, with 59 in the “no noise”' and 61 in the “noise” condition.

We found that live crowd noise across settings produced a difference in scores when compared with the judging with no noise. Using points awarded for the home fighter as the outcome measure (i.e., subtracting the away score from the home score), exposing judges to crowd noise resulted in a statistically significant difference of 0.53 points in favor of the home fighter. From a practical perspective, judges in different conditions awarded the same fight to different fighters in four of the thirty bouts (13.3%). In these bouts, judges in the noise condition awarded bouts to the home boxer, whilst judges in the no crowd noise condition awarded the bout to the away boxer. So actual outcome differences were seen in different crowd conditions when fights were closely contested.

Table 1 shows the scores awarded by judges in both conditions. The left hand column shows the range of point differences to the home competitor, the two central columns give the number of bouts that relate to those point differences, and the far right hand column just showing the total bouts with those point differences irrespective of condition. The shaded areas in the central columns show the 32 of the 120 scores (26.7%) where a change in noise condition could impact upon the result. The non-shaded areas (just under three-quarters of all decisions) highlight where a single point difference in favor of the home side could not impact upon the fight result, demonstrating limited practical importance (highlighting the important difference between practical and statistical significance).

**Table 1:**
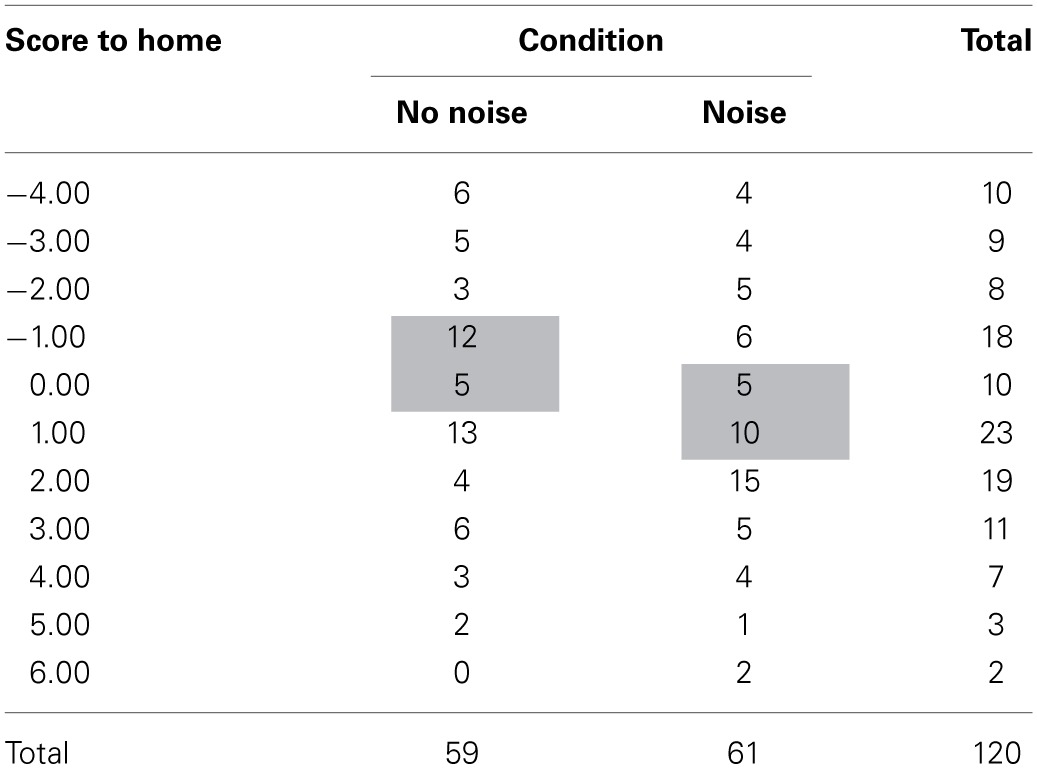
**Points in favor of the home boxer awarded by judges in the “noise” and “no noise” conditions (shaded areas indicate decisions where a change in the noise condition could impact on the result of the bout)**.

Close fights are fairly frequent in Muay Thai where boxers are matched closely for weight and experience. For example, Myers et al. (2010) using a larger dataset that included 405 individual judging decisions from 135 Muay Thai fights randomly selected in the UK and Thailand, found that in 158 of the judging decisions fighters were separated by a single point or less. Twenty-three of those decisions involved judges awarding even scores for both fighters. This suggests that crowd noise could have been a factor in 29.6% of the bouts as they were separated by a single point or less. Nevertheless, this is not to suggest all fights are closely contested. In the Myers et al. (2010) study judges applying Thai scoring criteria produced highly consistent decisions, but the actual points varied from drawn fights to 5-point differences (mean points difference = 2.3) between fighters (suggesting one fighter clearly won every round).

The findings of our study make an important contribution to the growing literature on the effects of crowds on sports officials and the influence on home advantage. The decisions awarded made an actual difference to the outcome of the competition and participants were aware of this. This is crucial to external validity. On one level the findings add to the body of literature on crowd influences on sport officials' decisions generally, but perhaps more importantly it offers a level of reassurance to the validity of the findings of previous laboratory experimental studies that had lower external validity (Nevill et al., 2002; Balmer et al., 2007; Unkelbach and Memmert, 2010). Our findings support the conclusion made by Anderson et al. (1999) following their comparison of effect sizes between laboratory and field studies where they suggested that “the psychological laboratory is doing quite well in terms of external validity; it has been discovering truth, not triviality” (p. 8).

There are strong arguments presented in the literature that internal threats to validity are far more important than considerations of those of external validity in theoretical research (Shadish et al., 2001). There is even encouragement for what was termed external invalidity in theoretical investigations (Mook, 1983). Nevertheless, we suggest the question of whether realism is an essential component of external validity when exploring theory largely depends on what question is being asked and the theories being tested. The live setting used in our study not only offered high external, internal validity and practical application, but also the possibility of a broader consideration of social theory explanations not possible with a laboratory setting. While some cognitive theories proposed to explain crowd effects can be satisfactorily investigated in a laboratory. For example, crowd noises used as a heuristic (a mental short cut) in the decision making process and cue-learning where noise is used a proximal cue to assess foul severity. Social conformity effects cannot be adequately explored in the same way when there is no possible participant-crowd interaction.

A real competition environment watched by an interactive live crowd that cares about decisions and vocalizes their feelings is very different from a recorded crowd in a laboratory. This real competition environment with a vocally responsive crowd offers the possibility of exploring social conformity effects not possible when there are no consequences to the decisions officials' make. Conformity is considered to be the result of either normative or informational influence (Deutsch and Gerard, 1955). As such, individuals conform either to be more accurate, for affiliation or to maintain positive self-concept (Cialdini and Goldstein, 2004). Cialdini and Goldstein (2004) argued that when accuracy is the motive for conformity, it is because the conformer believes others have cues for successful behavior. In the case of sports officials and crowd noise, this relates to the official considering the vocalized opinions of the majority of fans offering useful additional information to guide their decision. Conversely, normative conformity results from the conformer wanting to be accepted or valued by the group and demonstrating their agreement with the group or sport fans' views. We may add that sport officials may not just wish to conform just because of feeling valued by a partisan crowd, but arguably also because they feel intimidated by such a crowd. It is possible that both these forms of conformity influence judges' decisions. While both forms of conformity may be at play, we argue that normative conformity can only really be influential in experimental participants' decisions, where a live crowd is present and they perceive their decisions will be evaluated by others.

In Muay Thai, when there is a close fight it is possible that judges seek reassurance from the vocal majority in making judgment calls. However, equally, judges may have been swayed by perceived social sanctions from a passionate crowd after a decision is announced, or via “trial by web board” after the actual event in question. Judges' reputations have been subject to intense scrutiny in post-fight debates on the Internet. Certainly, in other contexts judges in sport have been shown to be influenced by conformity biases (Scheer et al., 1983; Vanden Auweele et al., 2004; Boen et al., 2006, 2008).

## Generalization

Given the range of different real settings used in our study combined with actual judges and live crowds, we feel confident that our findings generalize well across Muay Thai in the UK. This is particularly the case given the settings used included various sized venues, crowd sizes, crowd densities, and proximity to officials. We also feel the findings can be extended beyond Muay Thai to related sports such as Mixed Martial Arts (MMA) and professional boxing. These sports generally involve similar crowd composition, level of verbal support and involve ringside judges scoring bouts using a similar “ten point-must” system. Potentially, the effect could be magnified further in the case of professional boxing where bouts involve more rounds (up to 12). It appears crowd noise may in part explain the home advantage found in European championship boxing (Balmer et al., 2005). Equally, the effect of unusually large crowds generating extreme crowd noise may be more influential still. There is still the question to be answered as to “how much noise” or “how threatening” an environment needs to be to influence judges. Should we expect more bias in huge venues such as a sold out MGM grand in Las Vegas or Lumpinee stadium in Bangkok with a particularly partisan crowd?

## Future directions

Conducting a similar study to the one discussed here involving live crowds, but where comparisons are made of the potentially differing effects of different size venues may give a better idea of the important variables in the phenomena. Certainly this would go some way to address one concern with our live crowd study discussed. The “no crowd noise” condition involved the use of white noise. While this was useful in blocking out the crowd noise, it is not a sound that is naturally occurring within a judging context. The use of a “no crowd noise” condition meant it was not possible to determine whether crowd noise was used as a cue by judges to help determine the relative quality of blows delivered by competitors. Comparing the effect of crowd noise on different size venues may go some way to rectify this limitation, though of course it again limits internal validity by removing randomization and control over conditions. Unkelbach and Memmert (2010) postulated that differing volume and intensity of crowd noise may act as a cue to sports officials when determining magnitude, in similar ways to cue learning in perception (e.g., Jacobs, 2002), memory judgments (Unkelbach, 2006), and decision making (e.g., Evans et al., 2003). However, we acknowledge that even with these adjustments it would be difficult to separate cue learning effects from social conformity effects.

The use of noise-cancelling headphones in other sports offers a useful method of increasing external validity and a fruitful avenue of investigation. This would be easily applicable to sports where judges are stationary and sound is not a key component in the legitimate decision-making process, such as in gymnastics or ice-skating. These sports would offer an interesting insight into the influence of the crowd on aesthetic decision-making. As mentioned earlier, it would also be interesting to see if practical significance increases with greater volume or an increasingly partisan home crowd. Equally, making a comparison of the impact of differing levels of threat may help to separate public conformity from cue learning effects. While noise-cancelling headphones may be less practicable for mobile referees involved in sports such as basketball, football, and soccer, a possibility for future research could be the review decisions made in baseball, cricket, or rugby league.

In addition to refining our understating of crowd effects across sports, it would also be useful to identify or predict susceptibility to crowd effects in both individuals and judging systems. For example, different judging systems may well be influenced to different degrees by crowd effects. Muay Thai judging in Thailand has been found to be particularly consistent and more recently in the UK when similar systems were applied (Myers et al., 2010). Interestingly, in our live crowd study, it was the less experienced judges with limited formal training in the Thai judging system that identified different winners in three of the four bouts where this occurred. As such, experience and even the type of judging system employed may mediate against particular normative pressures. These types of investigation seem warranted from a practical perspective in combat sports, particularly given judging issues in Ultimate Fighting Championships (UFC) (Johnson, 2013). “Don't leave it in the hand of the judges” is never really an adequate situation for any sport.

Along with other researchers in this area, we have tended to discuss crowd noise as a single entity, considering differences in volume but little else. With the exception of examining booing and cheering (Greer, 1983) studies have generally neglected to investigate the content of crowd noise and its possible influence. Certainly in Muay Thai, there is anecdotal evidence that verbal utterances of boxing seconds and protests by high profile competitors may influence decisions disproportionally. These would be interesting future investigations. Finally, it would be also be interesting to investigate the inclination of coaches or managers to use video replay challenges in sports where they feel sports officials make inconsistent calls (as a result of home support or some other factor). This would help determine if coach or managers request an additional unbiased means to help correct any perceived imbalance.

## Conclusions

The studies reviewed here support the view that crowd noise can influence the decisions of sports officials. The results of laboratory experiments, observational, and archival findings combined with arguably first experimental evidence of the impact of live crowd noise on sport make a compelling case for this. However, the actual mechanism involved remains speculative, highlighting the need for further investigation. While there have been suggestions that external validity is of limited importance in theoretical investigations, we have argued for caution in this, and feel serious consideration needs to be given to external validity and representative design, particularly in applied research but also in selected theoretical contexts.

For those interested in reading more about home advantage in sport, the following articles provide excellent overviews: Allen and Jones (2014) provide a general overview of home advantage in athletic competition, providing a review of recent research on three conceptual models of home advantage, the Standard Model, the Territoriality Model, and Home Disadvantage; Jamieson (2010) offers a meta-analysis of home advantage findings across different sports, sports types (individual and team), time periods, length of season, and level of competition; Jones (2013) considers the question of whether home advantage in individual sports is comparable to that of team sports, and uses individual player quality in the assessing of home advantage for the individual sports considered.

### Conflict of interest statement

The author declares that the research was conducted in the absence of any commercial or financial relationships that could be construed as a potential conflict of interest.
